# A Novel Flushing Mechanism to Minimize Roughness and Dimensional Errors during Wire Electric Discharge Machining of Complex Profiles on Inconel 718

**DOI:** 10.3390/ma15207330

**Published:** 2022-10-20

**Authors:** Muhammad Umar Farooq, Saqib Anwar, M. Saravana Kumar, Abdullah AlFaify, Muhammad Asad Ali, Raman Kumar, Rodolfo Haber

**Affiliations:** 1 School of Mechanical Engineering, University of Leeds, Leeds LS2 9JT, UK; 2 Industrial Engineering Department, College of Engineering, King Saud University, P.O. Box 800, Riyadh 11421, Saudi Arabia; 3 Graduate Institute of Manufacturing Technology, National Taipei University of Technology, Taipei 10608, Taiwan; 4 Department of Industrial and Manufacturing Engineering, University of Engineering and Technology, Lahore 54890, Pakistan; 5 Department of Mechanical and Production Engineering, Guru Nanak Dev Engineering College, Ludhiana 141006, India; 6 Center for Automation and Robotics (CSIC-UPM), Spanish National Research Council-Technical University of Madrid, 28500 Madrid, Spain

**Keywords:** Inconel 718, complex geometry, machining, accuracy, surface integrity, dimensions, sustainability

## Abstract

One of the sustainability goals in the aeronautical industry includes developing cost-effective, high-performance engine components possessing complex curved geometries with excellent dimensional precision and surface quality. In this regard, several developments in wire electric discharge machining have been reported, but the influence of flushing attributes is not thoroughly investigated and is thus studied herein. The influence of four process variables, namely servo voltage, flushing pressure, nozzle diameter, and nozzle–workpiece distance, were analyzed on Inconel 718 in relation to geometrical errors (angular and radial deviations), spark gap formation, and arithmetic roughness. In this regard, thorough statistical and microscopical analyses are employed with mono- and multi-objective process optimization. The grey relational analysis affirms the reduction in the process’s limitations, validated through confirmatory experimentation results as 0.109 mm spark gap, 0.956% angular deviation, 3.49% radial deviation, and 2.2 µm surface roughness. The novel flushing mechanism improved the spark gap by 1.92%, reducing angular and radial deviations by 8.24% and 29.11%, respectively.

## 1. Introduction

For specific applications where conventional steels do not perform well, Inconel 718 is employed in the manufacturing and aerospace industries. The outstanding properties which make the alloy a first choice for aero-industry applications include superior endurance of mechanical properties at elevated temperatures, fatigue and corrosion resistance, higher creep strength, excellent engineering attributes, and an exceptional weight-to-thrust ratio [1,2]. This alloy is widely used by manufacturing industries because of its excellent thermo-mechanical functionality to make a variety of components for aircraft engines, including turbine discs, blades, combustors, and casings, as well as extrusion dies and containers, hot work tools, and dies [3,4].

**Figure 1:**
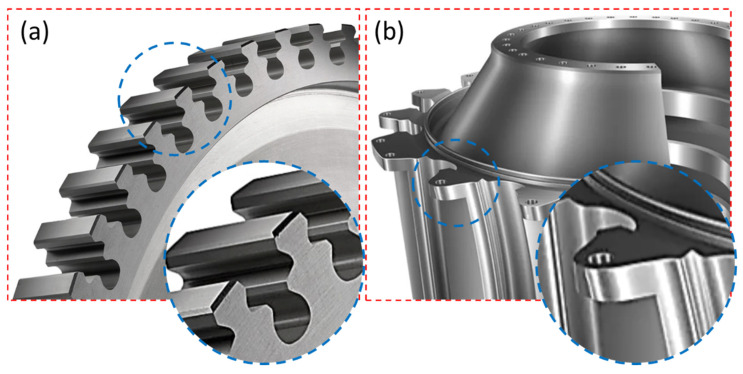
Application of complex profiles used in aero-industry: (**a**) turbine disk firtree profiles of Inconel 718 [6]; (**b**) fan disk of heat-resistant alloy [11].

Since the alloy is used for high-tech applications, it must be processed in several shapes as per functionality. Due to the alloy’s attractive physical properties, specific processing challenges are associated with it because of its hard-to-cut nature and poor thermal conductivity [5]. These challenges exponentially increase the machinability cost. There are several developments in conventional machining setups to overcome the cost barrier. These developments include customized lubrication systems, novel cutting tools, and thermally aided processing. However, these developments restrict production capabilities. Recently, the demand for the alloy has increased in hot structures and components of gas turbines and aero-engines, which are required as components with complex geometrical profiles [6]. Some examples where complex geometrical profiles are required include turbine discs with firtree profiles and fan discs, as shown in Figure 1. Therefore, the components’ dimensional accuracy and higher surface quality are critical for the application.

In the past decade, complex geometries have been processed through traditional machining processes. These conventional machining processes include turning, milling, and drilling. As the material possesses superior mechanical properties, the processing is limited because of tool wear, localized mechanical stresses at the surface and subsurface, and machinability challenges because of high hardness [7]. These limitations, along with poor production efficiency and performance, bound the machinability through conventional methods because of the thermo-mechanical properties of the alloy. Such problems limit productivity and push towards the consideration of non-conventional processes. 

Among non-conventional processes, wire electric discharge machining (WEDM) has gained popularity as an attractive alternative to process hard-to-cut materials with desirable quality attributes. Wire electric discharge machining (WEDM) is a variant of electric discharge machining die sinking with a replacement of fixed electrodes to continuously moving electrodes [8]. The primary function of the process is removing the material through successive electric discharges occurring at a certain frequency. The fundamental material removal mechanism is arguable to date. However, the basic understanding involves thermal conduction. This condition mechanism is administered by generating heat from spark channeling and dissipation in the tool electrode and workpiece. The discharge channels result in material melting and vaporizing, followed by flushing [9]. Therefore, the process is categorized into subtractive technologies for producing two- and three-dimensional features: through-drilling, irregular shape manufacturing, inclined hole making, and complex geometries. As mentioned earlier, the fabrication of the shapes is complicated by a single process on hard-to-cut alloy such as Inconel 718. 

However, the process is also used for surface texturing, enhancing surface hardness through the redeposition of electrode material as carbides, oxides, and surface alloying. In a nutshell, the process employs a thermo-electrical mechanism to machine the workpiece where sparking causes melting and evaporation of unwanted material. The tool electrode, which has a diameter of 0.05–0.30 mm, is a conductive wire which constantly moves by maintaining an inter-electrode gap of 10–100 μm [1]. Since there is no physical interaction between the tool and workpiece, the process is capable of machining any electrically conductive material of different dimensions [10]. However, the tool electrode is recommended to have higher thermal and electrical conductivity because of the involvement of high temperature while eroding the workpiece. Welling [3] made a comparison of surface quality and geometrical accuracy between wire electric discharge machining, grinding, and broaching for an Inconel 718 firtree-shaped (as shown in Figure 1a) jet engine component. In terms of geometric accuracy and overall surface integrity, the wire electric discharge machining process outperformed the conventional processes. The authors recommended the WEDM as a technologically advanced and attractive alternative for producing firtree profiles in Inconel 718. 

The literature on WEDM reveals that machining complex geometries is complicated because the wire electrode deviates from the programmed track, creating overcut and undercut errors. Therefore, the manufacturing community is carrying out significant focused research to overcome the limitations of fabricating complex geometries to meet the precision requirements of the aerospace industry. For instance, Farooq et al. [7] investigated the control of process parameters to obtain minimum geometric deviations and corner radii on Ti6Al4V. The authors machined complex curved profiles and obtained 0.250% overcut and 0.236% undercut errors in convex and concave shapes, respectively. In addition, servo voltage was considered the most controlling factor along with wire offset of 0.169–0.173 mm to attain 0.106 mm corner radii. Venkatarao and Kumar [12] analyzed the surface quality of Inconel 718 through wire tension, pulse current, pulse on time, and pulse off time. The authors concluded that wire displacement affects profile taper, and surface quality is controlled by wire tension and pulse current. Ahmed et al. [5] performed ultrafast drilling through electric discharge machining on Inconel 718. In the study, an additional DC power supply was integrated with the process to enhance the machining performance. The authors highlighted the significance of choosing the right tool electrode to improve the process efficiency. Dabade and Karidkar [13] optimized electric process parameters to machine complex geometries on Inconel 718, resulting in pulse on time having a significant impact on roughness and geometric deviations. In addition, the irregular discharge energy generated by servo voltage and pulse on time reduced the dimensional accuracy of the final part. Reolon et al. [9] investigated the process performance and surface integrity with coated and uncoated wire electrodes on Inconel 718. In the study, uncoated brass wire electrodes outperformed coated copper electrodes with a 36% increased feed rate and 80% reduced electrode consumption. The supremacy of the brass electrode was established through effective discharge energy transfer. Manoj and Narenderanath [14] machined triangular profiles having 0°, 15°, and 30° taper angles to study inaccuracy at Hastelloy X (nickel alloy). They found that insufficient flushing attributed to the generation of wire vibrations which increased slant angle inaccuracies (taper error). 

As the above literature shows, the wire electric discharge machine process depends on various factors such as workpieces, wire electrodes, dielectric attributes, process parameters, and geometric requirements. In addition, a trim-cut approach (limited to simple geometries) was applied to control the wire deflections originating from vibrations and discharge forces [15]. Similarly, Farooq et al. compensated wire offset while machining Ti6Al4V to minimize deflection, which causes wire lag [7]. Moreover, mathematical models have been developed to understand the discharge force and control through parametric optimization on low-radius free-form geometry [16]. Zahoor et al. [1] performed parametric optimization to minimize overcut and undercut through a genetic algorithm using coated electrodes on Inconel 718. Developments are underway to improve process capabilities regarding machining efficiency and better dimensional control. In this regard, different electrode materials and process optimization methodologies are opted to enhance surface integrity, reduce spark gap, and minimize dimensional errors, which are current limitations of the process. Ishfaq et al. [17] machined AISI D2 material to reduce geometrical machining errors incurred during die repair and maintenance. Maher et al. [18] highlighted challenges such as longer unattended operation periods, thicker workpieces, high taper angles, and complex profiles. The authors stressed effective flushing and cooling attributes to obtain higher machining performance. The cooling at the interface is important, as brass has a lesser melting temperature than Inconel 718, which introduces a cooling effect. This effect reduces the probability of wire breakage and enhances surface integrity. Zahoor et al. [1] machined Inconel 718 and found an optimized setting of servo voltage (54.62 V), wire tension (2.6405 g), and wire feed (4.17 mm/s), pulse on time (2.99 μs), and pulse off time (22.41 μs). The authors identified the necessity of an effective flushing mechanism with discharge energy to minimize geometric errors. Naveed et al. [19] machined curved profiles on WC-Co composite by employing wire tension (630, 730, 870 g), servo voltage (45, 50, 55 V), pulse-off-time (20, 25, 30 μs), and pulse-on-time (0.2, 0.3, 0.4 μs). The study resulted in a minimum of 6 μm radial error because the wire deflection originated from ineffective flushing.

Nayak and Mahapatra [20] investigated the effect of wire tension and speed, discharge current, pulse duration, taper angle, and part thickness on cryogenically treated Inconel 718. A minimum angular error was obtained through an optimized setting of wire tension (12 N), wire-speed (120 mm/s), discharge current (14 A), pulse duration (32 μs), and taper angle (5°). Similarly, Sharma et al. [21] improved the machining productivity and surface quality of Inconel 706 and compared several electrode materials. The study reported that zinc-coated electrodes enhanced cutting speed but deteriorated the surface integrity. However, the hard-brass electrode improved surface quality and outperformed in terms of lower residual stresses. In addition, the hard-brass electrode with effective control over discharge energy resulted in the potential to enhance the material removal rate. The supremacy of brass wire is established as a first choice to achieve higher surface quality. Klocke et al. [22] carried out parametric optimization with WEDM of fir tree slots in Inconel 718 and attained a 25% reduction in the recast layer and 40% productivity improvement. The literature mentioned above has established that different developments have been made to enhance geometrical accuracy and improve the surface integrity of complex profiles. In these approaches, the effect of flushing attributes on profile dimensional requirements is not studied compared to investigations on electrical parametric optimizations and the impact of electrode materials. To enhance machining performance, the aforementioned issues must be monitored and controlled. Furthermore, to strike a balance between machining efficiency, process stability, and geometric accuracy of the machined parts, flushing characteristics such as flushing pressure, flushing nozzle diameter, and flushing nozzle workpiece distance should be carefully controlled. The performance of WEDM during electro-sparks erosion is greatly influenced by controlled flushing, which involves precise flushing characteristics. Therefore, parametric evaluation of flushing pressure, flushing nozzle diameter, and flushing nozzle to workpiece distance might thereby improve the machinability in WEDM [23]. Optimized dielectric flushing influences the quality of the machined surface as well as processing speed. A high flushing pressure reduces machining speed, while the flushing nozzle to workpiece distance and flushing nozzle diameter increase wire vibrations and consequently wire lag, reducing the dimensional accuracy of the machined workpiece. Therefore, to increase machining efficiency, the wire lag should be decreased. According to Chakraborty et al. [24], an optimum combination of flushing features decreases the wire lag. It is well known that raising the flushing pressure and wire tension improves the component’s machinability and geometric accuracy. Electrical characteristics such as fine finish power supply, pulse width, servo voltage, and pulse current also play an essential role in improving machining performance, while enhanced debris removal is critical for stable machining. Based on the limited research that has been reported, Ehsan et al. [25] evaluated flushing attributes to enhance the material removal rate and surface integrity. The improved flushing action while machining M42 steel resulted in a 21.99 mm^3^/min material removal rate, 1.90 µm surface roughness, and 0.354 mm kerf width. Okada et al. [26] investigated nozzle jet flushing during the machining of SKD 11 and presented a reduced degree of wire deflection and breakage, while the debris residence time and flow fields were numerically evaluated against the distribution of hydrodynamic stress in the kerf. Roy and Sanna Yellappa [27] studied the effect of dielectric flushing conditions such as flushing pressure (0.5 to 1.5 kg/cm^2^) along with electrical machining parameters while processing (relatively simple geometry) TiNiCu alloy. The study showed that flushing pressure greatly influences surface morphology and flow direction significantly controls the removal of re-solidified debris and the amount of molten metal. Bergs et al. [28] analyzed workpiece to nozzle distance and flushing pressure as process variables to enhance the machinability of 16MnCr5 alloy. A multi-pass strategy was employed in the study to machine straight profiles for improved surface roughness and dimensional accuracy. An effective balance and more in-depth investigation were recommended in the study to correlate the flushing attributes to the quality matrix for useability in machining fir tree profiles on nickel alloys. Fujimoto et al. partially explored the essence of the flushing mechanism [29]. The authors employed jet flushing and stressed decreasing debris stagnation to rule out process instability, wire breakage, and shape inaccuracy.

It is evident from the literature survey that most studies focused on improving surface integrity, machining efficiency, and dimensional inaccuracies. However, the influence of flushing characteristics is not comprehensively studied, which could further be integrated into other developments, such as improved electrode coatings and better parametric conditions. In addition, the local industry uses WEDM systems with limited flexibility on flushing mechanisms. In this regard, the potentiality of wire electric discharge machining is explored to enhance surface integrity and reduce dimensional inaccuracies through a novel flushing mechanism. The experimental work focuses on maintaining dimensional precision on complex geometries in hard-to-cut Inconel 718. The complexity of the profile is derived from different features used in aero-engine applications. In this regard, complex shapes are assessed based on surface, subsurface damages, and dimensional capability (spark gap, angular error, and cylindricity error). The process is optimized by incorporating one electric variable servo voltage (as indicated in the literature to be the most significant in controlling erosion science) and three flushing variables of the system. The influences are quantified through analysis of variance, analyzed through micrographs, and scanning electron microscopy. In addition, it is paramount to highlight that the mono-objective optimization and multi-objective optimization of the complex profile processing are not carried out in the literature so far for the flushing attributes. An optimized parametric solution is developed to utilize the full potential of wire electric discharge machining coupled with a flushing mechanism by offering a tradeoff addressing the requirements of the aero-manufacturing industry. 

## 2. Materials and Methods

### 2.1. Experimental Setup

**Figure 2:**
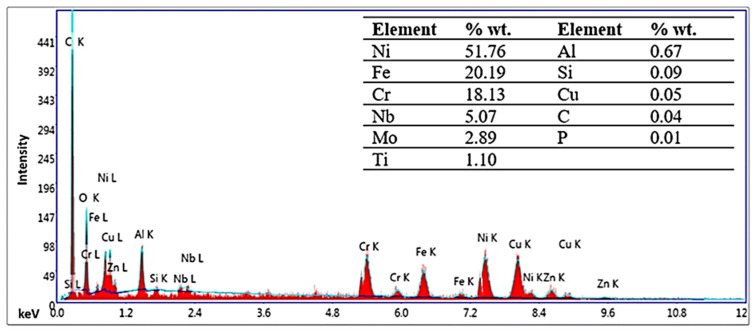
Energy-dispersive X-ray spectroscopy of the workpiece material, Inconel 718.

In this study, Inconel 718 is used as a workpiece to machine complex geometry profiles to evaluate the potentiality of the wire electric discharge machining process The composition of the workpiece is confirmed through energy-dispersive spectroscopy and compared with the manufacturer sheet, as shown in Figure 2. A spot-based method was used for energy-dispersive spectroscopy at 0.525 keV. The default settings were used to detect the peaks and elements based on the manufacturer’s recommendation, and Fe was present as a balancing element. Different physical and thermal properties that potentially influence the processing are mentioned in Table 1. 

The workpiece (of dimensions 147 mm × 50 mm × 8 mm (length × width × thickness)) was clamped using a customized fixture to avoid dimensional deviations. A constant clamping protocol was followed before each experiment, ensuring that the wire axis was parallel with the workpiece thickness. A continuously traveling brass wire electrode was used for experimentation because of the specific advantage of zinc in copper. The brass wire provides superior accuracy, cutting speed, and surface integrity compared to the copper wire electrode [9]. In addition, the brass (which has attractive properties because of 70% Cu and 30% Zn) decreases wire consumption remarkably with less maintenance. The selected wire electrode is superior because of the reduced deposition of powder and flaky debris on machine elements and improved cutting attributes. Moreover, it is established in the literature that brass wire outperformed all other variants in achieving higher surface integrity and lower residual stresses for Inconel material [21]. Therefore, a hard-type brass wire electrode having 900 MPa tensile strength and a diameter of 0.25 mm was selected. The properties of the wire electrode are mentioned in Table 1.

**Figure 3:**
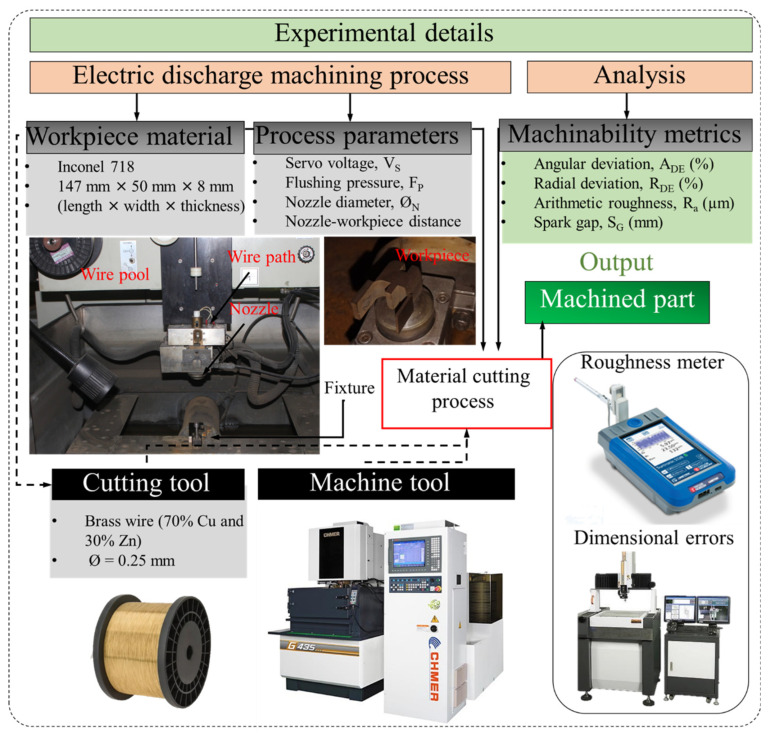
Experimental details and framework.

A machine tool, CNC wire EDM (CHMER G43S, Creator Taiwan), was utilized for experimentation (as shown in Figure 3 with other experimental details). De-ionized water was used as a dielectric, including a de-ionizing agent zeolite. A constant dielectric conductivity and concentration were ensured before the start of every experiment through regular monitoring. A storage tank of 590 L was used for experimentation to supply the dielectric; therefore, the thermal changes in the dielectric were not considered because of short machining time. The dielectric was constantly filtered out before reaching the tank.

**Figure 4:**
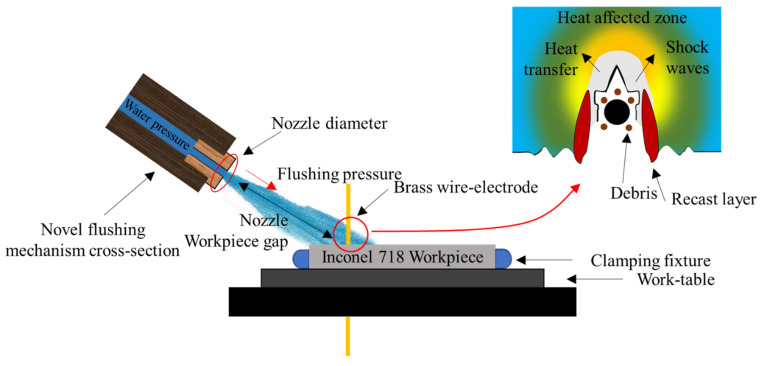
A novel flushing mechanism applied to the wire electric discharge machining process ([25] modified with permission from Springer).

The workpiece and wire electrode were immersed in the dielectric during the processing. An automatic wire feeder mechanism between the top and bottom nozzles threaded the wire to machine the workpiece. A customized novel flushing mechanism was manufactured to enhance the machining efficiency (as shown in Figure 4). In this mechanism, the flushing nozzle diameter is preferred to control flow rate and flushing pressure. Moreover, the nozzle plays a significant role in controlling the flushing action throughout the thickness of the workpiece, where the interface of the workpiece face and wire electrode interact. The nozzle flushes the dielectric to the erosion cavity. Three different sizes of nozzle diameters (4, 6, and 8 mm) were selected in this study. For each experimental condition, the nozzle of the required diameter was installed with a flushing mechanism. In addition, the distance between the flushing mechanism and the machining area was maintained and controlled effectively with the help of the manual pulse generator of WEDM to investigate the machining performance. 

The dielectric pressure is a machining parameter controlled through a variable knob. The significance of the dielectric flushing pressure is directly linked to the debris and molten metal removal. The distance alters the dielectric transfer and focus points during the erosion process. The wire electrode moves on the programmed path during the cutting action and produces the desired shape in the workpiece by generating a heat-affected zone and debris. Therefore, effective debris removal is controlled by the abovementioned flushing mechanism parameters. In addition, servo voltage is used to control the advancement and retraction of the wire electrode. This parameter helps to alter discharge energy transfer.

### 2.2. Experimental Design

Three-phase experimentation was carried out to investigate the potentiality of the flushing mechanism to minimize the process limitations. In the first phase, thorough trial experimentation was carried out to establish the effectiveness of the flushing mechanism. Efficient control over the mechanism and its correlation with machining action were studied thoroughly in the first phase. A problem encountered several times was wire breakage due to the complexity of the profile, which highlighted the need to manufacture a novel flushing setup. The range of flushing parameters was tuned, which remarkably reduced the wire breakage. This phase of experimentation was determined based on the reasonable parametric levels achieved through the developments and understandings from the first phase, as shown in Table 2. In the second phase of experimentation, Taguchi orthogonal array was considered for the design of experimental methodology to incorporate the statistical investigation of the process and to alleviate cost and time constraints. The Taguchi L_18_ design was chosen on two levels of servo voltage and three levels for flushing nozzle diameter, nozzle–workpiece distance, and flushing pressure. Three replicates of the second phase were performed to rule out the variability and statistical insignificance. Confirmatory experiments of optimal results were performed in the third phase.

The effects of the flushing mechanism were evaluated on different types of profile features. In the second phase of experimentation 0.125 mm offset was added in a programmed path to rule out the influence of the radius of the wire on geometrical accuracy. However, the resulting geometrical errors (angular and radial deviations) were found to be in the range of 9% to 16% of the designed dimensions. Therefore, a customized offset parameter was calculated for each parametric setting to further reduce the geometrical deviations, as shown in Equation (1). The complete design of experiments was repeated on the modified offset, and the results are analyzed herein.

(1) Wire offset=0.5×Wire diameter+Avg. geometric deviation        

### 2.3. Response Measurement

**Figure 5:**
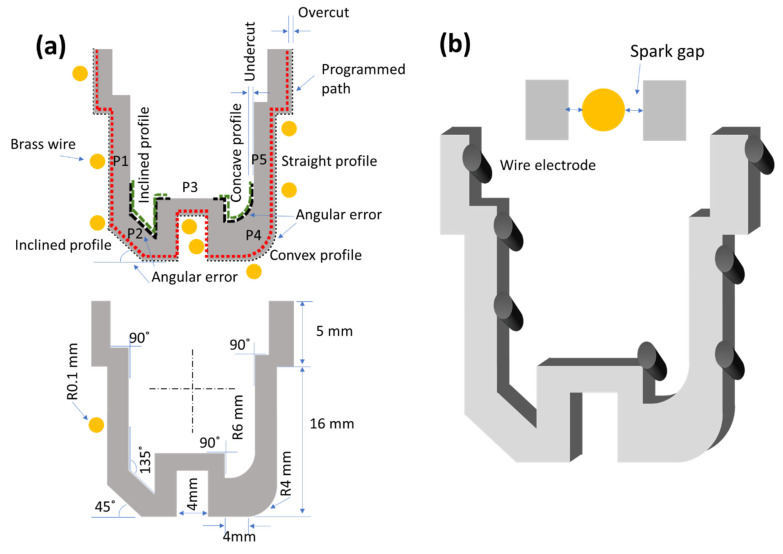
Details of the complex profile according to aero-engine requirements: (**a**) dimensions of machined profile; (**b**) 3D profile with an indication of the spark gap and wire orientation.

According to aero-engine requirements, a complex shape was selected, having curved, inclined, and straight features. The complete schematic and dimensions are illustrated in Figure 5 with a three-dimensional computer-aided design of the complex profile. A dedicated protocol was used to measure each response’s characteristics. 

Surface roughness was measured in different parameters, whereas arithmetic roughness *Ra* is widely used in the industry [31]. A surface texture meter (Surtronic S128, UK) was used to measure the arithmetic roughness *Ra*. A 0.8 mm cut-off and 4 mm evaluation length were followed to take measurements on the surface. Five dedicated places were selected to evaluate the effect of process variables on the profile. The P1, P3, and P5 positions showed straight profiles, whereas P2 showed an inclined profile and P4 illustrated a curved profile (convex and concave) in Figure 5. The roughness measurements were averaged after taking three values at each location and presented holistically. 

In terms of spark gap formation and angular and cylindricity errors, a coordinate measuring machining (Chen Wei CE 450 DV) was utilized (at 1 μm resolution) using a touch probe option to map radius and inclined geometries and a video camera option to measure the spark gap. The spark gap was measured using Equation (2) for n number of measurements at each experimental setting.

(2) $$S_{G}=\frac{1}{n}\overset{n}{\underset{i=1}{\sum }}\left[{\left(Measured\;kerf\;width\right)}_{i}-wire\;diameter\right]$$

For the measurement of angular and radial deviations, Equation (3) was used.

(3) $$for\;X_{DE}\left\{\begin{array}{c} A_{DE}\\ R_{DE} \end{array}\right.\;X_{DE}=\frac{1}{n}\overset{n}{\underset{i=1}{\sum }}\left[\frac{Programmed\;geometric\;dimensions-{\left(Measured\;geometric\;dimensions\right)}_{i}}{Programmed\;geometric\;dimensions}\times 100\right]$$

The differences in the angle and radius are illustrated in Figure 5 with the dotted line. The surface morphology was investigated through microscope OLYMPUS STM6-LM. The key characteristics of the experimental data are shown in Table 3. 

## 3. Results and Discussion

### 3.1. Parametric Significance Analysis

Analysis of variance (ANOVA) was carried out to define the parametric significance of output responses. The influence of the parameters was compared between each parametric level and among repetitions of each experiment. The adequacy of the models has been confirmed through mean and standard deviations under confidence intervals. The summarized results of the F-value and *p*-value are shown in Table 4. The *p*-value is assessed against the confidence interval of α = 0.05. The α helps draw the parametric significance by showing a 95% confidence interval. The process parameter having a low *p*-value as compared to α is categorized as a significant variable in the response. Moreover, all the input control variables are compared, and the significance is analyzed based on the above-mentioned criteria. 

Table 4 shows that servo voltage and nozzle–workpiece distance were not found to be significant on surface roughness compared to other variables. The reason behind this behavior is that the range of process parameters is short enough to record any significant effect on the surface roughness. However, flushing pressure (*p*-value < 0.001) and flushing nozzle diameter (*p*-value < 0.001) showed a substantial impact on surface roughness. On the other hand, in the case of spark gap formation, all process parameters showed a significant effect (*p*-value < 0.001). At the same time, the flushing nozzle diameter showed weak significance as its *p*-value < 0.041 is close to the α-value of 0.05. Similarly, in the case of angular and radial deviations, all process parameters have significantly affected the errors. However, in the case of angular deviation, the nozzle–workpiece distance has shown weak significance (*p*-value 0.047), which slightly relates the process phenomenon with discharge energy generation. 

In addition to the *p*-value, which determines the significance of the parameter, percentage contribution is another factor that helps to characterize the proportion among variables to influence the response [32]. The percentage contribution (PCR) ratio helps comprehensively prioritize the process parameters’ relevance to affect output responses. In this regard, PCR is calculated by the adjusted sum of squares (Adj SS) and the total sum of squares (Total SS), as shown in Equation (4).

(4) $$\mathrm{Percentage}\;\mathrm{contribution}\;\left(\mathrm{PCR}\right)=\left[\frac{\mathrm{Adj}\;\mathrm{SS}}{\mathrm{Total}\;\mathrm{SS}}\times 100\right]\%$$

**Figure 6:**
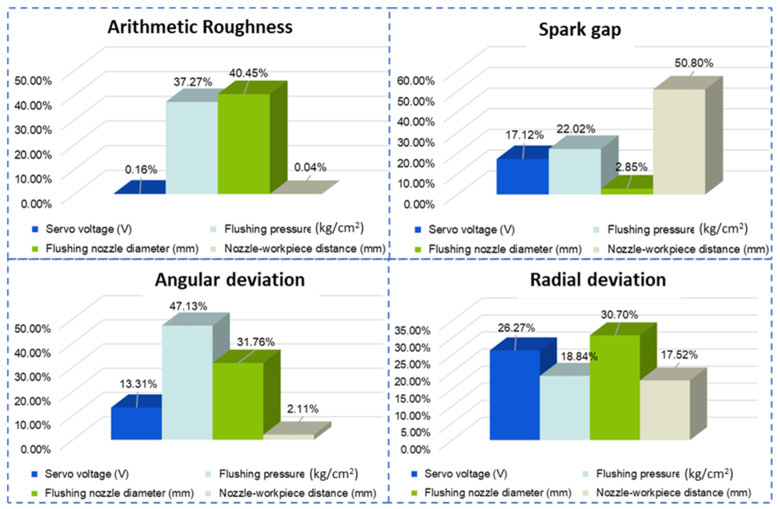
Percentage contribution ratio of WEDM process parameters in controlling the responses.

The percentage contribution ratio against each output response is plotted in Figure 6. Flushing nozzle diameter and flushing pressure majorly controlled the balance of discharge energy and flushing action by 40.45% and 37.27%, respectively. In addition, angular deviation during the machining of complex profiles on Inconel 718 was controlled by flushing pressure (47.13%), flushing nozzle diameter (31.76%), and servo voltage (13.31%). In the same analogy, control variables influenced spark gap formation in balance. However, the variables related to the flushing mechanism affected the formation of the spark gap the most. The nozzle–workpiece distance was the top variable with 50.80% to control kerf width, followed by flushing pressure (22.02%) and servo voltage (17.12%). The control by the flushing mechanism is associated with an effective evacuation of debris and balanced flushing–melting synergy. In addition, the radial deviation was controlled by variables in balance. The most control was with flushing nozzle diameter (30.70%), followed by servo voltage (26.27%), flushing pressure (18.84%), and nozzle–workpiece distance (15.52%). A thorough statistical investigation highlights the necessity of an effective flushing mechanism design. Figure 6 illustrates that flushing nozzle diameter and pressure are the most influential parameters in machining efficiency, surface quality, and dimensional accuracy. Therefore, a balanced and optimized approach is required to select the variables mentioned above to utilize the wire electric discharge machining process capability efficiently. 

### 3.2. Process Optimization

#### 3.2.1. Mono-Objective Optimization

In this research, single-objective optimization is carried out through signal-to-noise ratio as supported by Taguchi analysis to define the optimal parametric settings. Through this methodology, a signal response is considered objective at a time and optimized against “larger the better”, “nominal the better”, and “smaller the better” goals. These goals assist to determine the validatory experiments and improve the response compared to the set of experiments. Since the current study incorporates angular and cylindricity errors, spark gap formation, and arithmetic roughness, the “smaller the better” approach was used to determine the signal-to-noise ratios [33]. The mathematical equations that help determine the signal-to-noise ratio are provided by Equations (5) and (6).

(5) $$\eta ij=-10log[\frac{1}{n}\overset{n}{\underset{K=1}{\sum }}(Yij^{2}\;)]$$

(6) $$\eta ij=-10log[\frac{1}{n}\overset{n}{\underset{k=1}{\sum }}(\frac{1}{Yij^{2}\;})]$$

In both of the equations, *Y_ij_* is the average response value that is under consideration. The count of *n* repetitions of the experiments is represented by *n*. The signal-to-noise ratio comparison of parametric levels against each response is shown in Table 5. 

In Table 5, rank determines the influence of the particular process variable on the output response. Moreover, the quality of the response on each parametric level is determined by the signal-to-noise ratio magnitude, which should be higher. A higher signal-to-noise ratio among parametric levels assures the supremacy of the level to obtain desired results. In this way, optimal parametric settings are determined against each response. 

The confirmatory experimentation results in Table 6 highlight the sovereignty of the novel flushing mechanism and its settings in improving the spark gap by 1.92%, reducing angular and radial deviations by 8.24% and 29.11%, respectively. The optimized settings for arithmetic roughness include V_S_ 1_,_ F_P_ 3, Ø_N_ 1, and W_D_ 2 levels. Similarly, the optimal parametric levels for spark gap are V_S_ 1_,_ F_P_ 1, Ø_N_ 1, and W_D_ 1. The angular deviation is reduced significantly by using V_S_ 1_,_ F_P_ 3, Ø_N_ 1, and W_D_ 3 parametric levels. Lastly, a radial deviation is minimized by using V_S_ 1_,_ F_P_ 1, Ø_N_ 3, and W_D_ 1 optimal settings. 

#### 3.2.2. Multi-Objective Optimization Using Grey Rational Analysis 

The sensitivity of the process affirms that a slight deviation in the parametric level introduces a complicated synergistic influence on the process behavior [34]. Therefore, an ideal achievement of each goal at the same time is difficult to realize. In this regard, single-response optimization significantly compromises the effectiveness of the process in other directions [35,36]. On the other hand, multi-objective optimization introduces a better compromise by finding an optimized solution in terms of conflicting responses [37]. The responses such as arithmetic roughness, spark gap formation, and angular and radial deviation need to be jointly optimized to present an effective solution of the flushing mechanism for better performance. 

In this work, grey relational analysis, a multi-objective optimization approach, optimizes conflicting responses statistically. The approach can potentially validate various parameters by avoiding deficiencies in statistical analyses. The sequence of the actual response values is normalized from 0 to 1 based on the nature of the intended response. The normalization is carried out using objectives such as “higher the better”, as evident in Equation (7); “nominal the better”, as mentioned in Equation (8); and “lower the better”, as highlighted in Equation (9).

(7) $$\mathrm{Higher}\;\mathrm{the}\;\mathrm{better}\;\to X_{ij}=\frac{Y_{ij}-{\left(Y_{ij}\right)}_{min}}{{\left(Y_{ij}\right)}_{max}-{\left(Y_{ij}\right)}_{min}}$$

(8) $$\mathrm{Nominal}\;\mathrm{the}\;\mathrm{best}\;\to X_{ij}=1-\frac{\left|Y_{ij}-Y\right|}{{\left(\beta_{ij}\right)}_{max}-\beta }$$

(9) $$\mathrm{Lower}\;\mathrm{the}\;\mathrm{better}\;\to X_{ij}=\frac{{\left(Y_{ij}\right)}_{max}-Y_{ij}}{{\left(Y_{ij}\right)}_{max}-{\left(Y_{ij}\right)}_{min}}$$

In the above equations (Equations (6)–(8)), the *j*th experiment in the *i*th sequence is illustrated through the normalized measure $X_{ij}$. The *i*th response value of the *j*th experiment is shown by $Y_{ij}$. Similarly, the desired output measure is shown as Y. The responses such as arithmetic roughness, spark gap formation, and angular and radial deviations are jointly optimized based on the “lower the better” objective, as mentioned in Equation (10). Grey relational coefficient (GRC), as mentioned in Equation (9), is used to correlate the normalized values with the optimal response values.

(10) $$\gamma_{ij}=\frac{{\left(\Delta \right)}_{min}+\xi {\left(\Delta \right)}_{max}}{\Delta_{ij}+\xi {\left(\Delta \right)}_{max}}$$

The grey relational coefficient of the *i*th normalized measure of the *j*th experiment is shown by $\gamma_{ij}$. The coefficient has a value between 0 and 1 and is characterized as a differentiating coefficient. The variable ξ = 0.5 is used for this study for multi-response optimization. Moreover, $\Delta_{min}$ shows minimum absolute deviation (as shown in Equation (11)) from the target. Similarly, $\Delta_{max}$ shows maximum absolute deviation (as shown in Equation (12)) from the target. Likewise, the divergence of normalized measure from target values is determined through a deviation sequence described as $\Delta_{ij}$, which is calculated using Equation (13).

(11) $$\Delta_{min}=min_{i}min_{j}\left(y_{i}^{o}-y_{ij}\right)$$

(12) $$\Delta_{max}=max_{i}max_{j}\left(y_{i}^{o}-y_{ij}\right)$$

(13) $$\Delta_{ij}=\left(y_{i}^{o}-y_{ij}\right)$$

The grey relational coefficients are summed in a weighted manner (which is equal in this case) to compute the grey relational grade (GRG). The grey relational grade connects all response measurements and input variables to present an optimized solution holistically.

(14) $$\delta_{j}=\frac{1}{n}\sum_{i=1}^{n}\gamma_{ij}$$

The grey relational grade of the *j*th experiment (Equation (14)) is indicated by $\delta_{j}$. The number of responses is indicated by *n*. The grey relational grade varies between 0 and 1, where the highest magnitude shows the optimality of the parametric settings to achieve the desired outcome simultaneously. The rank is defined based on the intra-grade comparison. The detailed methodology and calculations are indicated in Table 7. 

In Table 7, the optimized parametric settings indicated by grey relational grade are V_S_ 50 V, *F_P_* 4 kg/cm^2^, *Ø_N_* 8 mm, and *W_D_* 10 mm. The parametric settings show an optimal compromise between all other responses and achieve the target by 70.8%. The grey relational analysis affirms that the selected parametric levels produce optimal conditions simultaneously. The result of the confirmatory experiment reduced the process’s limitations to a 0.109 mm spark gap, 0.956% angular deviation, 3.49% radial deviation, and 2.2 µm surface roughness. 

### 3.3. Process Control Analysis

#### 3.3.1. Arithmetic Roughness

**Figure 7:**
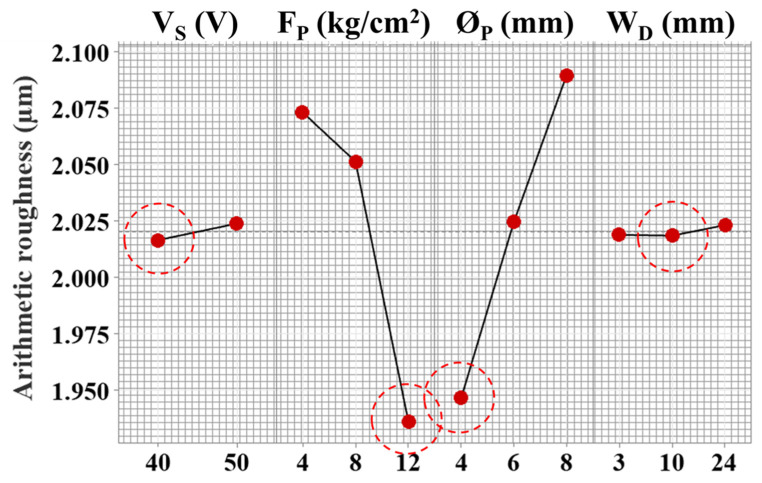
Parametric effects on surface roughness of complex profile machining and red circles indicating desired parametric level.

The surface roughness measures surface discrepancies generated from the electro-thermal erosion process, and it is an essential feature in machining processes [38,39]. The machined surface features are a function of various factors such as discharge energy, dielectric flushing, and material properties. The machining parametric effects are illustrated in Figure 7. The influence of the novel flushing mechanism is characterized by analysis of means where the mean of each response is taken at each level to compare the performance holistically. 

**Figure 8:**
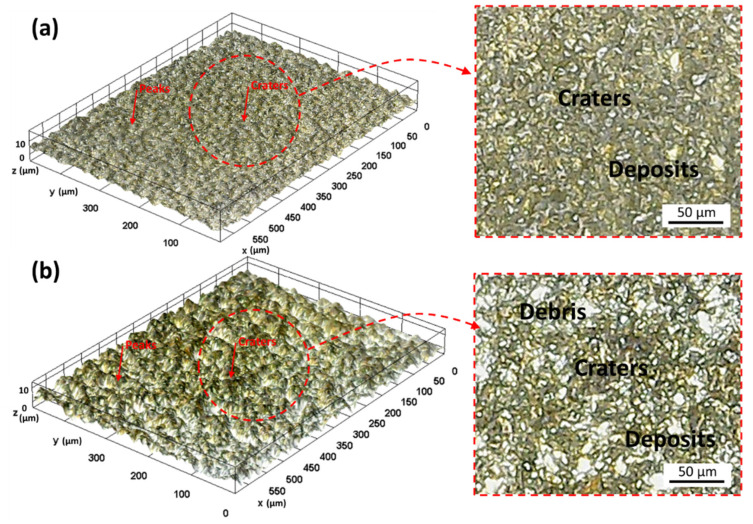
Surface micrographs (**a**) V_S_ 40, F_P_ 12, Ø_N_ 4, W_D_ 10 (low discharge energy); (**b**) V_S_ 50, F_P_ 4, Ø_N_ 4, W_D_ 8 (high discharge energy).

The servo voltage has a direct relationship with roughness and depicted around 2 µm. The servo voltage controls the interelectrode gap between the wire and the workpiece. The higher servo voltage is linked with the enhancement of the interelectrode gap. The increased gap promotes redeposition of the debris, which disturbs heat flux. Therefore, a roughness of ~2.025 µm was recorded from the compromised discharge energy transfer (considered fundamental to material erosion in the process) and unbalanced material removal [25]. The wire vibrations and deflection are reduced at lower servo voltage, providing improved surface quality with the lower crater, cracks, and roughness, as shown in Figure 8a. However, the chance of wire breakage increases with lower servo voltage values because there is less space between the wire electrode and the workpiece. Increased discharge energy and spark intensity are the causes of wire breakage. Because wire breakage commonly happens as a result of slight variations, it is a highly important component in setting the parameter’s range [7]. The nozzle–workpiece distance variable affects surface roughness in an increasing fashion. In addition, the effect of servo voltage and nozzle–workpiece distance is not significant enough compared to flushing pressure and nozzle diameter. 

On the other hand, surface quality was improved by increasing the flushing pressure, as Roy and Sanna Yellappa [27] highlighted the influence of flushing attributes such as pressure (0.5 to 1.5 kg/cm^2^) significantly affecting surface morphology. At low flushing pressure of 4 kg/cm^2^, the roughness is around 2.075 µm, which decreased by ~7% by increasing the flushing pressure to 8 kg/cm^2^ (Figure 7). At low flushing pressure, the primary liquid flow principle results in compromised melt and debris removal, which increases the surface roughness, where low flushing pressure contributes to the high density of craters, leading to higher roughness. The surface morphology is a significant quality indicator of parametric effects. In Figure 8a, low servo voltage and high flushing pressure result in low discharge energy and an effective cooling and debris removal action. However, in Figure 8b, high servo voltage and low flushing pressure are attributed to high discharge energy and poor melt removal. It is illustrated in the micrographs (Figure 8) that a lower flushing quality caused melted debris and other distinctive attributes. The prominent features such as debris re-solidification, craters, and cracks result from the discharge energy phenomenon, which helps remove unwanted material. The poor flushing translates into the generation of surface irregularities on machined profiles. The discharge energy produces a melt pool, which is fundamentally required to be removed, and low flushing pressure (4 kg/cm^2^) agglomerates the debris, resulting in a coarser surface. However, with the increased flushing pressure to 6 kg/cm^2^, surface quality improved and resulted in ~2.05 µm roughness. The slight increase in flushing pressure balances the spark gap to provide cleaning and cooling rates to the interelectrode interaction area. In addition, a further rise in the flushing pressure of the dielectric to 8 kg/cm^2^ minimizes the contamination of the working region between the electrodes and the surface being machined of complex profiles machining and does not let contaminants affect current flow. According to Ehsan et al. [21], low flushing pressure contributes to generating a resistance path, which is detrimental to surface integrity through the arcing effect. 

The low flushing pressure of the dielectric fluid is directly related to higher roughness values because it is associated with the low degree of cooling effect in the workpiece–electrode interaction area. The low cooling effect is translated into greater heat generation, accelerating the material evaporations (Figure 8). Fundamentally, the high evaporation of material and discharge energy require a higher degree of flushing action to reduce the redeposition of debris on the machined surface. Therefore, an effective flow and replacement of the dielectric in the interaction area are essential to reduce the craters. Zahoor et al. [1] stressed the effective removal of hot debris from the machined surface in curved features of profile. The authors also linked the adversity of the flushing mechanism with heat generation, which contributed to higher surface roughness. 

Efficient control of the parametric settings is required to minimize surface defects and improve surface quality. In this regard, the nozzle diameter linearly affects surface roughness, as shown in the parametric effects plot (Figure 7). With the increase in nozzle diameter from 4 mm to 6 mm, surface roughness declined by ~4%, showing ~2.025 µm. At a lower diameter, the flushing mechanism is focused on the interaction area. However, with the increase in diameter, the dedicated flushing action volume increased by introducing different process dynamics. As Ehsan et al. [21] discussed, a smaller nozzle diameter introduces the possibility of vapor film generation on the wire, increasing the cooling effect. Therefore, this contributes to a reduction in surface roughness. Conclusively, a lower nozzle diameter is recommended to improve surface integrity. However, using an effective flushing mechanism has improved the surface integrity by several folds on straight and complex geometries. The main cause of cooling in WEDM is dielectric fluid evaporation. However, there is a chance that a vapor film will form on the wire and workpiece, which will lessen the subsequent cooling and dry-out condition since the vapor film prevents liquid from touching the wire and serves as an insulation layer. If it is not possible to completely eliminate the vapor layer, it should be reduced in thickness to promote cooling. By boosting the dielectric’s pressure and flow rate via the nozzles, this might be accomplished. Therefore, greater lubricant mobility is needed to cool the contact region and improve debris flushing. As a result, high flushing pressure and small nozzle diameter potentially reduce the surface roughness. 

#### 3.3.2. Spark Gap Formation

**Figure 9:**
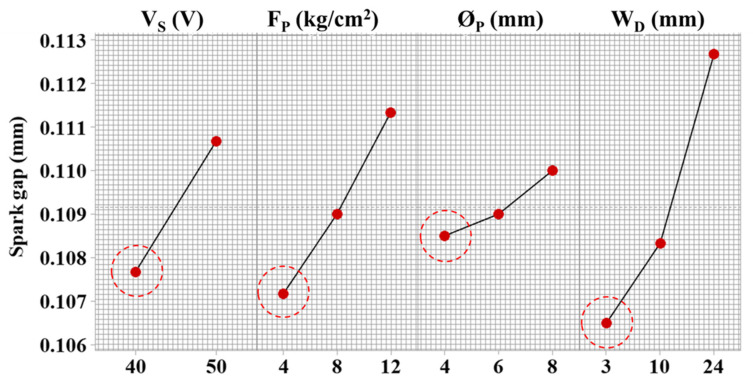
Parametric effects on spark gap formation of complex profiles machining and red circles indicating desired parametric level.

The spark gap formation represents the excessive removed material along the wire path, which is a limitation of the electro-spark erosion process. However, the adequacy of material removed with a reduced spark gap is a desirable objective. The main effect plots are shown in Figure 9, where parametric effects are characterized quantitatively. A linear relationship of servo voltage is evident with spark gap formation. With the increase in servo voltage from 40 V to 50 V, the spark gap increased from 107.5 µm to 111 µm, showing a ~3.2% upsurge. The fundamental science behind this behavior is related to the inter-electrode gap. Since the spark gap is formed in all directions of the wire, an unbalanced melting behavior removes excessive material in all directions of the workpiece interaction area. This concludes in an increase in erosive action translated into higher sparking and material removal (because of poor thermal conductivity of 11.2 W/m K). Similarly, higher servo voltage prolongs the spark duration because of an increased spark gap. A similar process science is endorsed by Farooq et al. [7].

Comparing the parameters controlling the flushing mechanism, an increasing trend is visible where the spark gap increased with the increase in the magnitude of variables. The flushing pressure of 4 kg/cm^2^ results in a 107 µm spark gap. With the increase in flushing pressure up to 12 kg/cm^2^, the spark gap increased significantly to 111.5 µm (Figure 9). Therefore, a small flushing pressure helps to remove adequate material and presents a balanced energy transfer. The increased spark gap with the higher flushing pressure is linked to faster immature melt removal (because of poor thermal conductivity of Inconel 718 (11.2 W/m K)), resulting in an over-material melting phenomenon [29]. The wire electrode generates an electric sparking channel, which increases the temperature and causes melting on the surface. Due to high flushing pressure and nozzle diameter (which increases the dielectric flow to the interaction area), excessive material removal is carried out, which rules out the balance between geometric details to be machined and actual machined geometry [25]. Therefore, the spark gap increased with the dielectric flow volume, which affects melt removal, as the poor thermal conductivity of Inconel 718 (11.2 W/m K) does not let the energy dissipate in the workpiece. Therefore, the increased energy results in increased melt volume, which is quickly removed by the flushing action. In the next frequency cycle, the same process repeats, resulting in excessive removed material. Similarly, an increase in nozzle diameter from 4 mm to 8 mm resulted an increase in spark gap from 108.5 µm to 110 µm (Figure 9). Likewise, the workpiece distance with nozzle enhanced immature removal of melt and introduced irregularities on the surface by introducing deeper craters [27]. With the increase in distance from 3 mm to 24 mm, the spark gap increased from 106 µm to 112.5 µm, showing a 6% increase, as depicted in Figure 9. 

**Figure 10:**
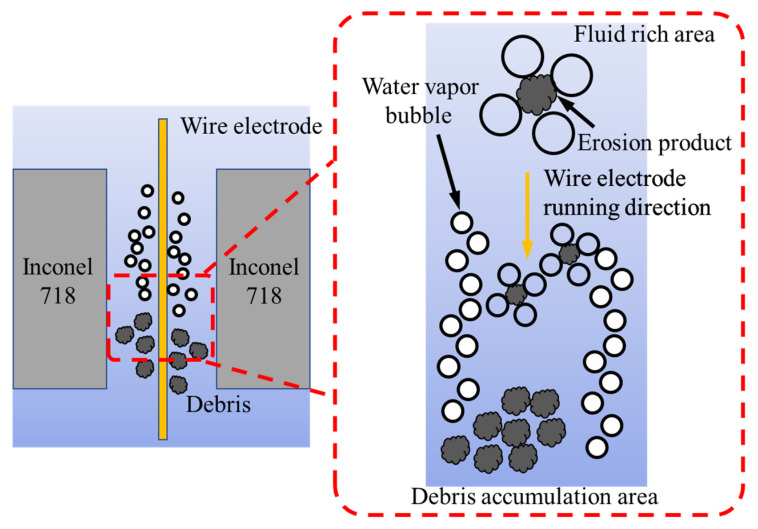
The cutting mechanism affecting process efficiency through spark generation, debris removal, and discharge gap.

With the increase in the nozzle–workpiece gap, the volume of flushing action increases, adversely affecting the erosion process performance. The cutting mechanism where effective flushing is required is shown in Figure 10. A poor flushing action reduces the vapor film formation on the wire outer surface, which potentially provides a cooling effect as brass has a lower melting point of 940 °C as compared to Inconel 1260–1336 °C. Therefore, high flushing pressure, distance, and dielectric volume cause local dry-out conditions, resulting in compromised dielectric mobility in the interface. A similar process science is endorsed by Wang et al. [40]. On the other side, the very low magnitude of flushing pressure and other flushing characteristics result in the accumulation of debris (as illustrated in Figure 10) during the machining of complex profiles. The debris creates a resistance band and affects the erosion process. This mechanism supports the need to employ a dedicated and optimized flushing mechanism to avoid the dry-out condition, effectively flushing in the interface, and minimize debris accumulation. 

#### 3.3.3. Angular Deviation

**Figure 11:**
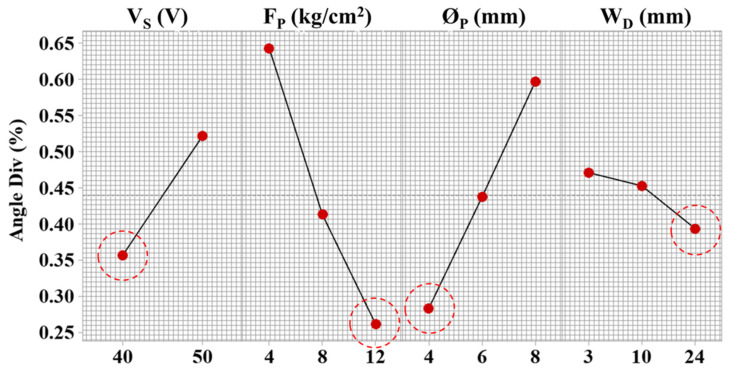
Parametric effects on an angular error of complex profiles machining and red circles indicating desired parametric level.

The parametric effects on the angular deviation during the machining of Inconel 718 are illustrated in Figure 11. The analysis is based on the means at each parametric level against the angle deviation from designed geometry to machined geometry. 

The servo voltage shows an increasing linear trend on angle deviation. The spark voltage governs spark intensity and adequacy of discharges through effective control of advancement and retraction of the wire electrode. The deviation increased from 0.35% to 0.53% by increasing servo voltage from 40 V to 50 V (Figure 11). At a large voltage magnitude, the instability of spark strength affected the energy transfer and adequacy of melt pool generation. This instability results in inconsistent machined cavities with compromised dimensional details. A similar process science is discussed by Ishfaq et al. [41] on geometrical errors during Al6061 machining. Therefore, efficient control over discharge energy and its frequency is required to achieve lower angular deviations. 

**Figure 12:**
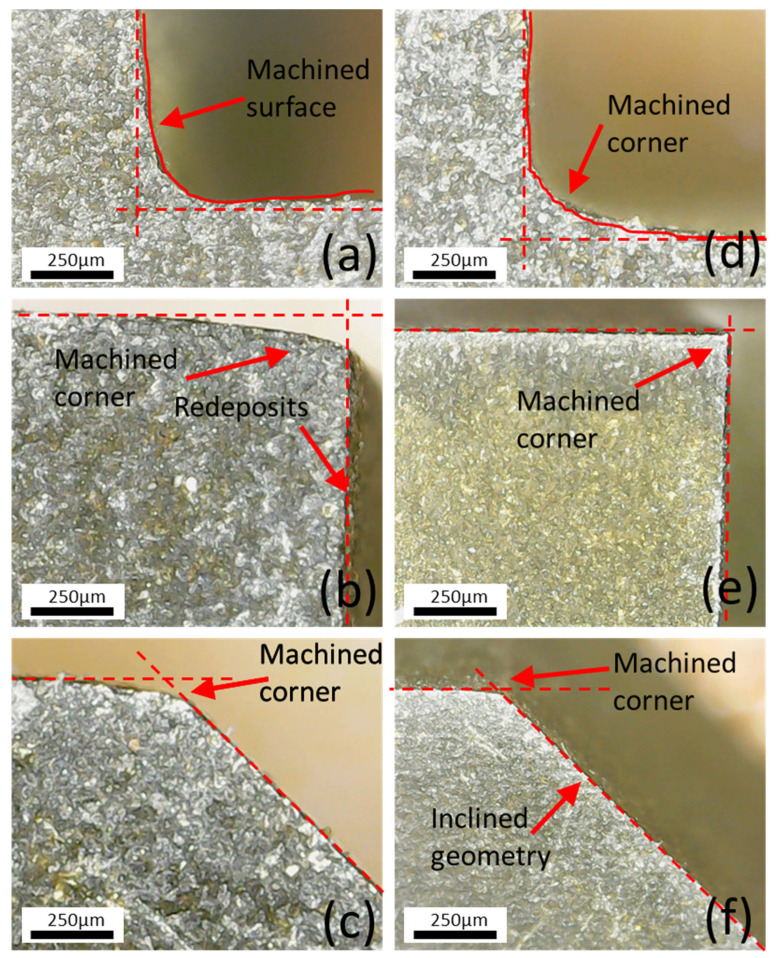
Angular deviation and corner formation: (**a**–**c**) V_S_ 40 V, F_P_ 12 kg/cm^2^, Ø_N_ 4 mm, and W_D_ 10 mm (un-optimized); (**d**–**f**) V_S_ 50 V, F_P_ 4 kg/cm^2^, Ø_N_ 8 mm, and W_D_ 10 mm (optimized).

On the other hand, the increase in flushing pressure from 4 kg/cm^2^ to 12 kg/cm^2^ decreased the angle deviation from 0.65% to 0.25% (Figure 11). The decrease in deviation is attributed to reduced amplification of wire vibrations. In addition, the electro-erosive forces are generated during the electric sparking, which removes the adequate material. An increased flushing pressure increases the removal of melt material from the surface and does not let debris accumulate, as shown in Figure 9. Secondly, the force inserted on the moving wire by the injected dielectric facilitates controlling the deviations from design geometry [25]. The angular deviations are displayed in Figure 12. The right-angle profile shows a corner formation in both conditions (optimized and un-optimized), as shown in Figure 12a,d. Similarly, another right-angle profile (shown in Figure 12b) indicates comparatively accurate results at optimal settings of flushing attributes as compared to poor flushing and energy transfer (shown in Figure 12e). Likewise, the inclined plane offers an efficient fabrication of the corner and reduced angular deviation with effective flushing attributes using optimized settings (shown in Figure 12f) in comparison to un-optimized settings (shown in Figure 12c). 

With the increase in nozzle diameter from 4 mm to 8 mm, deviation increased from 0.255% to 0.6% (Figure 12). The high nozzle diameter increases entry of dielectric flow, which hinders the stability of the thermo-electric erosion process near corners and tends to alter sparking orientation because of the inoculation of the dielectric in the interface. At a higher nozzle diameter, dielectric transfer to the interaction area is more, which introduces local wire lagging because of deflection from programmed geometry. Thus, it generates the destabilization of gap voltage and sparking. In addition, the generation of heat flux is reduced, introducing higher dimensional deviation at corners. The increased wire tension results in higher fluctuations and wire breakage during machining [16]. With the increase in nozzle–workpiece distance from 3 mm to 24 mm, angle deviation reduced by up to 0.4% (Figure 11). 

#### 3.3.4. Radial Deviation

**Figure 13:**
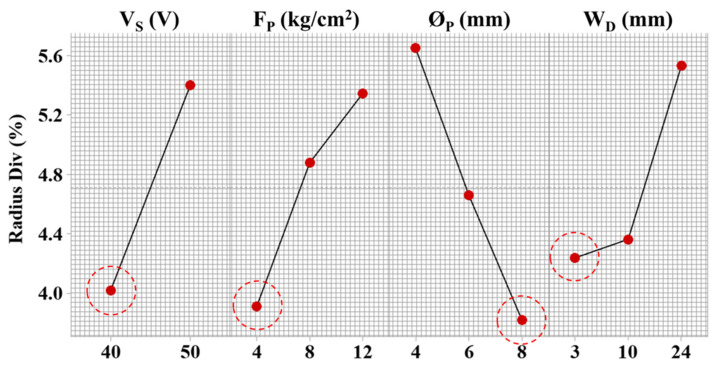
Parametric effects on cylindricity error of complex profiles machining and red circles indicating desired parametric level.

Similar parametric behavior is observed in radial deviation to a higher degree as compared to angular deviation. For instance, with the increase in servo voltage from 40 V to 50 V, the radial deviation increased from 4% to 5.4% (Figure 13). A higher degree of deviation is generated because the curved profiles carry a complex mechanism of machining. The wire electrode acts as a tangent at every point on the convex or concave feature. Therefore, the high discharge energy flux required to perform melting and vaporization change direction at every point of travel. The changing direction of sparks induces a thermal pool at the surface, which is integrated with the workpiece because of having a poor thermal conductivity of 11.2 W/m K. Because of inadequate heat dissipation, the redeposition of the debris, and their accumulation in a larger spark gap (at higher servo voltage), the cutting rate is compromised, which affects the dimensional accuracy. A similar process science related to the spark gap is discussed by Sharma et al. [21].

The flushing pressure shows an increasing linear trend with the radius deviation. At 4 kg/cm^2^, the radius deviation is around 4%, which increased to 5.4% at 12 kg/cm^2^ (Figure 13). The influence of process parameters is more on radius deviation than angular deviation. A low degree of deviations is experienced in straight and inclined geometries as compared to curved profiles. The prime reason behind higher deviation at convex and concave profiles is wire deflection, which reduces the repetitive discharge sparking in the interaction area. Therefore, this unbalanced sparking affects the flexibility and wire electrode mobility dynamics. Moreover, the thickness of the workpiece plays an important role in minimizing the deflections by an effective selection of drive speed. The dielectric volume could minimize the stretching effect because of the discharge forces [28]. At a higher nozzle diameter of 8 mm, a large dielectric volume is transferred to the interaction area, which minimizes the effects of unbalanced sparking and maximizes melt removal action by decreasing re-solidification and accumulation of debris. 

**Figure 14:**
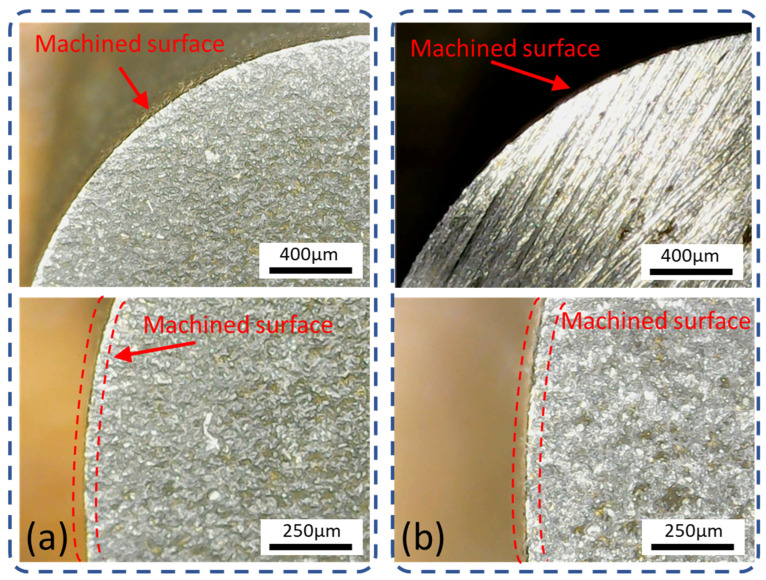
Radial deviation: (**a**) V_S_ 40 V, F_P_ 4 kg/cm^2^, Ø_N_ 6 mm, and W_D_ 10 mm (un-optimized); (**b**) V_S_ 50 V, F_P_ 4 kg/cm^2^, Ø_N_ 8 mm, and W_D_ 10 mm (optimized parameter).

The machined surface, which shows radial deviation at experimental (un optimized) conditions and optimal parametric settings indicated by GRA, is shown in Figure 14a,b. From Figure 14, it is clear that the radial deviation at the optimal setting was less than the un-optimized condition. The deviations at the edge are because the effect of discharge force is inevitable, as discussed by Ishfaq et al. [41]. In poor flushing conditions, heat dissipation is reduced with reduced debris removal. The gap introduces non-uniform and uneven spark distribution, resulting in losing control over spark intensity. The material exclusion, unflushed droplets, and melted debris redeposit on the surface and disturb the geometry through undercut deviations [7]. Moreover, effective melting action on anodic workpiece polarity with the promotion of local melting integrated with an over-flushing control induces overcut deviations [1]. Therefore, a balanced flushing action and discharge energy transfer are required to obtain the richness of the process in achieving dimensional accuracy of convex and concave profiles. 

### 3.4. Scanning Electron Microscopic Analysis of Machined Surface

**Figure 15:**
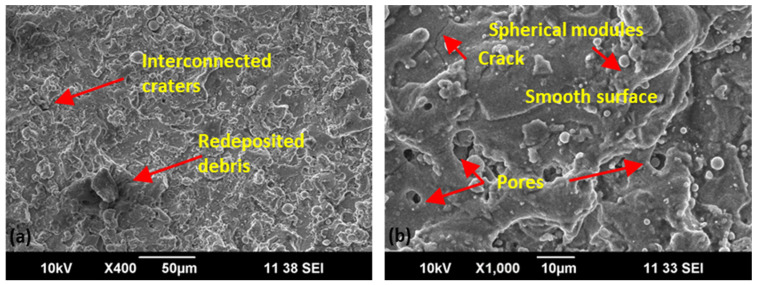
Scanning electron microscopic analysis of straight geometry for optimal parametric setting (V_S_ 50 V, F_P_ 4 kg/cm^2^, Ø_N_ 8 mm, and W_D_ 10 mm) at ×400 (**a**) and ×1000 (**b**).

**Figure 16:**
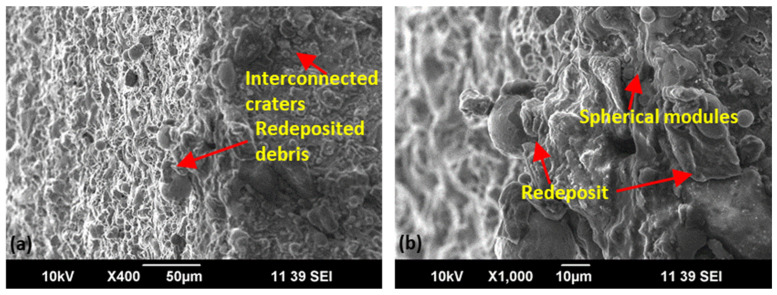
Scanning electron microscopic analysis of concave geometry for optimal parametric setting (V_S_ 50 V, F_P_ 4 kg/cm^2^, Ø_N_ 8 mm, and W_D_ 10 mm) at ×400 (**a**) and ×1000 (**b**).

**Figure 17:**
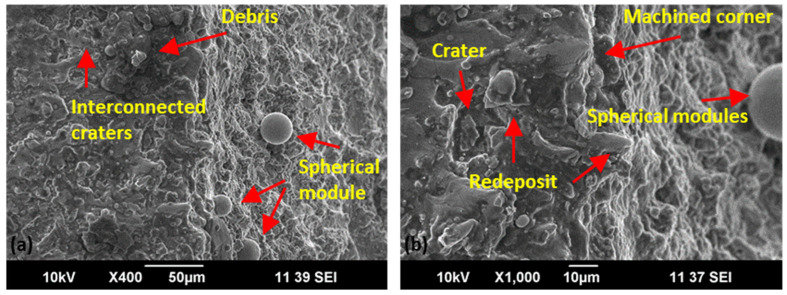
Scanning electron microscopic analysis of convex geometry for optimal parametric setting (V_S_ 50 V, F_P_ 4 kg/cm^2^, Ø_N_ 8 mm, and W_D_ 10 mm) at ×400 (**a**) and ×1000 (**b**).

Detailed scanning electron microscopic analysis of the machined surface of straight, concave, and convex geometry for optimal parametric setting (V_S_ 50 V, F_P_ 4 kg/cm^2^, Ø_N_ 8 mm, and W_D_ 10 mm) indicated by GRA was conducted at X400 and X1000 (Figure 15, Figure 16 and Figure 17). A relatively smooth straight profile was observed at optimal settings, as shown in Figure 15. A smoother surface with a low intensity of cracks, interconnected craters, and redeposited debris affirms the supremacy of the flushing mechanism on straight profiles resulting in Ra = 2.2 μm, as evident in Figure 15.

The discharge energy transfer caused by the interconnected craters, spherical modules, and redeposited debris is observed at concave geometry in Figure 16. The surface analysis highlights the thermo-electrical erosion mechanism where different surface features are produced. The interconnected craters, smooth surface, and less degree of the spherical module are evident in the concave geometry. The generation of surface discrepancies significantly depends on the discharge energy transfer and the material’s thermo-electrical properties. As shown in Figure 16, the interconnected craters result from balanced energy transfer and flushing action.

It is evident in Figure 17 that discharge energy controls the surface characteristics. Moreover, complex geometries increase the chances of debris accumulation because of the complexity of the machining path [22]. Therefore, the stiffness of the wire electrode, balanced energy transfer, adequate melt pool generation, and a higher degree of flushing throughout the interaction area of the wire face and workpiece thickness are needed to improve the surface features of the complex profiles. However, Figure 17 shows the redeposition of debris and melted deposits with interconnected craters on the convex feature of the complex profile (in the minimal proportion which endorses the optimality of the process). An effective flushing action safeguards by removing debris, with less degree of surface irregularities, a balanced distribution of discharge energy by removing carbon from the interaction area, and yields a better surface finish [41]. Moreover, the dielectric performs an immediate quenching effect on the heat-affected zone, which changes the surface’s mechanical properties. Craters, cracks, redeposits, and spherical modules are visible in Figure 17 at optimized flushing settings due to improved cooling rate and efficient debris removal. A reduced degree of debris adhesion is visible on the surface, which affirms the effectiveness of the flushing mechanism. A similar material behavior is endorsed by Zahoor et al. [1], and similar thermal energy transfer and flushing action science are endorsed by Ehsan et al. [25]. 

Since curved profiles are being used in aero-engine structures, achieving complex shapes such as curved profiles and inclined and straight profiles through an effective alternative for hard-to-cut materials such as wire electric discharge machining is challenging because of overcutting and undercutting issues. Considering the tolerance requirements of the aeronautical industry, in the current study, we attempted to achieve minimum dimensional errors resulting from deviations from programmed geometry. The pivotal role of the novel flushing mechanism is investigated through parametric effects where higher control over geometrical requirements and surface integrity could be achieved through an optimized setting. 

## 4. Conclusions

In the experimental work, a dedicated focus is established to upkeep the dimensional precision on complex geometries at hard-to-cut Inconel 718. Therefore, complex profiles are assessed based on surface integrity (arithmetic roughness and surface morphology) and dimensional capability (spark gap, angular error, and cylindricity error). The process is optimized by incorporating one electric variable servo voltage (as indicated in the literature to be the most significant in controlling erosion science) and three flushing variables of the system. As per thorough analysis, the following conclusions are drawn.

- A high degree of error is experienced in curved profiles (generating a radial error) compared to straight and inclined geometries (generating an angular error). The influence of process parameters is more significant on radius deviation as compared to angular error. At 4 kg/cm^2^, the radius deviation is around 4%, which increased to 5.4% at 12 kg/cm^2^.
- The low flushing pressure (4 kg/cm^2^) agglomerates the debris, which resulted in a coarser surface because of the re-solidification of debris with a surface roughness of 2.12 µm. However, with the increase in flushing pressure to 8 kg/cm^2^, surface quality improved and resulted in ~1.93 µm roughness.
- An increasing trend is observed where the spark gap increased with the magnitude of flushing variables. The flushing pressure of 4 kg/cm^2^ resulted in a spark gap of 107 µm. With the increase in flushing pressure up to 12 kg/cm^2^, the spark gap increased significantly to 111.5 µm. Similarly, an increase in nozzle diameter from 4 mm to 8 mm increased the spark gap from 108.5 µm to 110 µm.
- The change in nozzle diameter from 4 mm to 8 mm increased angular error from 0.255% to 0.6%. The high nozzle diameter increased the entry of dielectric flow, which hindered the stability of the thermo-electric erosion process near corners.
- The flushing pressure (*p*-value < 0.001) and flushing nozzle diameter (*p*-value < 0.001) showed a significant effect on the surface roughness. On the other hand, in the case of spark gap formation, all process parameters showed a significant effect (*p*-value < 0.001), whereas the flushing nozzle diameter showed weak significance as its *p*-value < 0.041 (close to the α-value of 0.05).
- The optimized parametric settings indicated by grey relational grade are V_S_ 50 V, *F_P_* 4 kg/cm^2^, *Ø_N_* 8 mm, and *W_D_* 10 mm. The confirmatory experiment reduced the process’s limitations to a 0.109 mm spark gap, 0.956% angular error, 3.49% cylindricity error, and 2.2 µm surface roughness.

As EDM is an electro-thermal process, it depends on the material’s intrinsic properties, which limits the study to a specified material. The study is limited to performance evaluation and does not incorporate all factors of surface integrity. It will be interesting to evaluate how generated surfaces (having different characteristics) perform under tribological assessment matrix where the fatigue and reliability are major points of concern.

## Figures and Tables

**Table 1:** Physical properties of workpiece and wire electrode [30].

Properties	Inconel 718	Brass (70% Cu and 30% Zn)	Units
Melting point	1260–1336	940	°C
Electric conductivity	8 × 10^5^	16 × 10^6^	S/m
Specific heat capacity	0.435	0.380	J/g K
Thermal conductivity	11.2	115	W/m K
Density	8.19	8.73	g/cm^3^

**Table 2:** Process control variables and response characteristics.

Input Control Variables						Response Characteristics		
		Parametric Levels						
Machining parameter	Symbol	Level 1	Level 2	Level 3	Units	Response variables	Symbol	Units
Servo voltage	*V_S_*	40	50	-	V	Arithmetic roughness	*Ra*	µm
Flushing pressure	*F_P_*	4	8	12	kg/cm^2^	Spark gap	*S_G_*	mm
Nozzle diameter	*Ø_N_*	4	6	8	mm	Angular deviation	*A_DE_*	%
Nozzle–workpiece distance	*W_D_*	3	10	24	mm	Radial deviation	*R_DE_*	%

**Table 3:** Response characteristics.

Characteristics	*Ra* (µm)	*S_G_* (mm)	*A_DE_* (%)	*R_DE_* (%)
Max	0.116	0.956	8.400	2.120
Min	0.104	0.109	2.610	1.010
Avg.	0.109	0.439	4.709	1.419
Std. dev.	0.004	0.227	1.350	0.509

**Table 4:** Parametric significance analysis through ANOVA.

Arithmetic Roughness		
Input Variable	F-Value	*p*-Value
Servo voltage (V)	0.09	0.763
Flushing pressure (kg/cm^2^)	21.95	<0.001
Flushing nozzle diameter (mm)	23.82	<0.001
Nozzle–workpiece distance (mm)	0.03	0.874
R^2^ = 77.92%; R^2^ (adj) = 71.13%		
Spark Gap		
Input Variable	F-Value	*p*-Value
Servo voltage (V)	30.93	<0.001
Flushing pressure (kg/cm^2^)	39.77	<0.001
Flushing nozzle diameter (mm)	5.15	0.041
Nozzle–workpiece distance (mm)	91.75	<0.001
R^2^ = 92.80%; R^2^ (adj) = 90.59%		
Angular Deviation		
Input Variable	F-Value	*p*-Value
Servo voltage (V)	30.41	<0.001
Flushing pressure (kg/cm^2^)	107.66	<0.001
Flushing nozzle diameter (mm)	72.55	<0.001
Nozzle–workpiece distance (mm)	4.82	0.047
R^2^ = 94.31%; R^2^ (adj) = 92.56%		
Radial Deviation		
Input Variable	F-Value	*p*-Value
Servo voltage (V)	51.22	<0.001
Flushing pressure (kg/cm^2^)	36.74	<0.001
Flushing nozzle diameter (mm)	59.86	<0.001
Nozzle–workpiece distance (mm)	34.17	<0.001
R^2^ = 93.33%; R^2^ (adj) = 91.28%		

**Table 5:** Mono-optimization through the signal-to-noise ratio.

(a) Arithmetic Roughness				
Level	V_S_	F_P_	Ø_N_	W_D_
1	−6.083	−6.325	−5.783	−6.097
2	−6.114	−6.236	−6.120	−6.086
3		−5.735	−6.393	−6.113
Delta	0.031	0.589	0.610	0.026
Rank	3	2	1	4
(b) Spark gap				
Level	V_S_	F_P_	Ø_N_	W_D_
1	19.36	19.40	19.30	19.46
2	19.12	19.26	19.26	19.31
3		19.07	19.17	18.97
Delta	0.24	0.33	0.12	0.49
Rank	3	2	4	1
(c) Angular deviation				
Level	V_S_	F_P_	Ø_N_	W_D_
1	10.132	4.222	12.020	7.704
2	6.668	8.486	8.104	8.543
3		12.491	5.075	8.952
Delta	3.464	8.269	6.944	1.248
Rank	3	1	2	4
(d) Radial deviation				
Level	V_S_	F_P_	Ø_N_	W_D_
1	−11.83	−11.48	−14.75	−12.42
2	−14.40	−13.66	−13.09	−12.50
3		−14.21	−11.51	−14.42
Delta	2.56	2.73	3.24	2.00
Rank	3	2	1	4

**Table 6:** Mono-objective optimized confirmatory experiments.

Response Measures	DOE Data		SN Ratio Data		% Improvement from DOE Results
	Un-Optimized Settings	Response Values	Optimized Settings	Confirmatory Experiments Results	
*Ra* (µm)	V_S_ 1_,_ F_P_ 3, Ø_N_ 1, W_D_ 2	1.863	V_S_ 1_,_ F_P_ 3, Ø_N_ 1, W_D_ 2	1.863	-
*S_G_* (mm)	V_S_ 2_,_ F_P_ 1, Ø_N_ 2, W_D_ 1	0.104	V_S_ 1_,_ F_P_ 1, Ø_N_ 1, W_D_ 1	0.102	1.92%
*A_DE_ (%)*	V_S_ 1_,_ F_P_ 3, Ø_N_ 1, W_D_ 2	0.182	V_S_ 1_,_ F_P_ 3, Ø_N_ 1, W_D_ 3	0.167	8.24%
*R_DE_ (%)*	V_S_ 1_,_ F_P_ 1, Ø_N_ 2, W_D_ 2	2.61	V_S_ 1_,_ F_P_ 1, Ø_N_ 3, W_D_ 1	1.85	29.11%

**Table 7:** Multi-objective optimization using grey rational analysis.

Exp No.	Normalizing Sequence				Grey Relational Coefficient				Grey Relational Grade	Rank
	*R_DE_*	*S_G_*	*A_DE_*	*Ra*	*R_DE_*	*S_G_*	*A_DE_*	*Ra*		
1	1.000	0.642	0.834	0.723	0.333	0.438	0.375	0.409	0.389	18
2	0.833	0.470	1.000	0.170	0.375	0.516	0.333	0.746	0.492	8
3	0.500	0.358	0.934	0.269	0.500	0.583	0.349	0.650	0.520	6
4	1.000	0.913	0.620	0.607	0.333	0.354	0.446	0.452	0.396	16
5	0.917	0.741	0.724	0.626	0.353	0.403	0.409	0.444	0.402	15
6	0.333	0.629	0.720	0.226	0.600	0.443	0.410	0.688	0.535	5
7	0.667	1.000	0.573	1.000	0.429	0.333	0.466	0.333	0.390	17
8	0.250	0.901	0.504	0.780	0.667	0.357	0.498	0.391	0.478	11
9	0.750	0.723	0.902	0.500	0.400	0.409	0.357	0.500	0.416	14
10	0.500	0.580	0.392	0.667	0.500	0.463	0.561	0.429	0.488	10
11	1.000	0.171	0.646	0.428	0.333	0.745	0.436	0.539	0.513	7
12	0.583	0.000	0.848	0.000	0.462	1.000	0.371	1.000	0.708	1
13	0.583	0.732	0.430	0.654	0.462	0.406	0.538	0.433	0.460	12
14	0.083	0.620	0.423	0.343	0.857	0.446	0.542	0.593	0.610	3
15	0.583	0.215	0.734	0.191	0.462	0.699	0.405	0.724	0.573	4
16	0.000	0.901	0.000	0.864	1.000	0.357	1.000	0.367	0.681	2
17	0.417	0.774	0.579	0.777	0.545	0.392	0.464	0.391	0.448	13
18	0.250	0.625	0.610	0.781	0.667	0.445	0.451	0.390	0.488	9

## Data Availability

The raw or processed data required to reproduce these findings cannot be shared at this time as the data also form part of ongoing work.
